# Residual Tensile Property of Plain Woven Jute Fiber/Poly(Lactic Acid) Green Composites during Thermal Cycling

**DOI:** 10.3390/ma9070573

**Published:** 2016-07-14

**Authors:** Hideaki Katogi, Kenichi Takemura, Motoki Akiyama

**Affiliations:** Department of Mechanical Engineering, Kanagawa University, 3-27-1 Rokkakubashi, Kanagawa-ku, Yokohama, Kanagawa 221-8686, Japan; takemura@kanagawa-u.ac.jp (K.T.); r201470067pd@jindai.jp (M.A.)

**Keywords:** green composite, natural fiber, PLA, residual tensile property, thermal cycling, coefficient of linear expansion, fiber pull out

## Abstract

This study investigated the residual tensile properties of plain woven jute fiber reinforced poly(lactic acid) (PLA) during thermal cycling. Temperature ranges of thermal cycling tests were 35–45 °C and 35–55 °C. The maximum number of cycles was 10^3^ cycles. The quasi-static tensile tests of jute fiber, PLA, and composite were conducted after thermal cycling tests. Thermal mechanical analyses of jute fiber and PLA were conducted after thermal cycling tests. Results led to the following conclusions. For temperatures of 35–45 °C, tensile strength of composite at 10^3^ cycles decreased 10% compared to that of composite at 0 cycles. For temperatures of 35–55 °C, tensile strength and Young’s modulus of composite at 10^3^ cycles decreased 15% and 10%, respectively, compared to that of composite at 0 cycles. Tensile properties and the coefficient of linear expansion of PLA and jute fiber remained almost unchanged after thermal cycling tests. From observation of a fracture surface, the length of fiber pull out in the fracture surface of composite at 10^3^ cycles was longer than that of composite at 0 cycles. Therefore, tensile properties of the composite during thermal cycling were decreased, probably because of the decrease of interfacial adhesion between the fiber and resin.

## 1. Introduction

Interior components of scrap vehicles have been recycled to comply with end-of-life vehicle recycling laws in Europe [1] and Japan [2]. However, the interior components of scrap vehicles, which are produced using petroleum-based resin, present great difficulty for recycling. Therefore, green composites [3,4,5,6,7,8,9,10] are being emphasized for use as new generation components of vehicles. Green composites are fabricated using natural fiber and biodegradable polymer resin. Among biodegradable polymer resins, poly(lactic acid) (PLA) is mainly used as the matrix. The life cycle assessment of the PLA [11] is smaller than that of petroleum resins such as polypropylene, polyester, and polyvinyl chloride. The specific Young’s modulus of natural fiber as reinforcement is similar to that of glass fiber. The specific gravity of natural fiber is about 1.4 [3].

Recently, numerous reports have described mechanical properties [12,13,14,15,16,17,18,19,20] and molding method [21,22,23,24,25] of green composite. Toyota Boshoku Co., Ltd. (Kariya, Aichi, Japan) produced door trim ornaments using the green composite [26]. The interior components of vehicles are used at environmental temperatures. In August 2012 in Japan, the average environmental temperature in vehicles was about 51 °C [27]. Therefore, many reports of the relevant literature describe effects of environmental temperatures on tensile properties of the green composites.

Ben et al. [28] described tensile properties of kenaf fiber reinforced PLA at environmental temperatures. Tensile strength and Young’s modulus of kenaf fiber reinforced PLA under 50 °C decreased 27% and 75%, respectively, compared with those of kenaf fiber reinforced PLA at room temperature (RT). Nassiopoulos et al. [29] reported an effect of environmental temperature on tensile properties of flax fiber reinforced PLA. Tensile strength and Young’s modulus of flax fiber reinforced PLA under 110 °C decreased 62% and 79%, respectively, in comparison with those of flax fiber reinforced PLA under RT. Katogi et al. [30] described some flexural properties of jute fiber reinforced PLA under environmental temperature. Flexural strength and Young’s modulus of jute fiber reinforced PLA under 50 °C decreased 22% and 12%, respectively, in comparison with those of jute fiber reinforced PLA at RT.

Interior components of vehicle are used under thermal cycling that occurs each day. In addition, environmental temperatures related to weather change each day. Effects of thermal cycling on tensile properties of green composites under some temperature ranges should be investigated for long-term use. In Japan and Europe, vehicle inspections must be conducted about once every two years [31]. Therefore, some reports of the literature describe effects of thermal cycling on tensile properties of natural fiber reinforced composites using petroleum resin. Sakiyama et al. [32] reported effects of thermal cycling on tensile properties of jute fiber reinforced polypropylene. For temperatures of −20–80 °C, the Young’s modulus of jute fiber reinforced polypropylene at 20 cycles was lower than that of jute fiber reinforced polypropylene at 0 cycles.

However, few studies have examined the effects of thermal cycling on tensile properties of green composites as a bioresource-based interior component. This study investigated tensile properties of green composites during thermal cycling. Thermal properties and tensile properties of the matrix and reinforcement were also investigated after thermal cycling.

## 2. Materials and Molding Method

Plain woven jute fiber (1717, Yasuda Co., Ltd., Arakawa-ku, Tokyo, Japan) knitting jute spun yarn was used as reinforcement. PLA (Ecodea 300-1B01DA, Toray Co., Ltd., Chuo-ku, Tokyo, Japan) was used as the matrix. The molding method of green composites was vacuum compression molding, with temperature and pressure of 190 °C and 1.7 MPa, respectively. The molding time was 10 min. Subsequently, the molding temperature was cooled to RT. The fiber volume fraction of green composites was 40%.

The molding method used for PLA was vacuum compression molding. The molding temperature was 190 °C. The molding pressure was 0.4 MPa. The molding time was 10 min. Subsequently, the molding temperature was cooled to RT.

**Figure 1:**
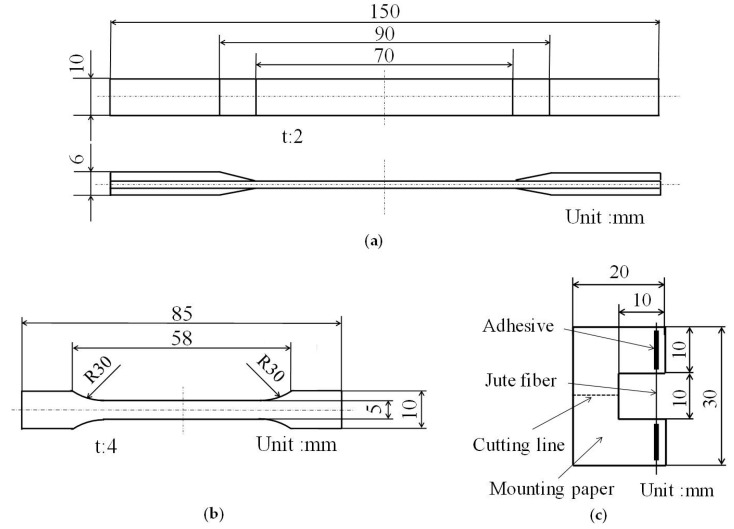
Schematic drawing of specimens: (**a**) Green composite; (**b**) Poly(lactic acid) (PLA); (**c**) Jute fiber.

Jute fiber consists of lignin (12%–13%), hemicellulose (14%–20%), and cellulose (61%–71%) [33]. Elementary fiber bound jute fiber has longitudinal variation in its cross sectional area. The specific gravity of jute fiber is 1.3 [5]. Figure 1 presents a schematic drawing of specimens.

## 3. Testing Method

### 3.1. Thermal Cycling Test

**Figure 2:**
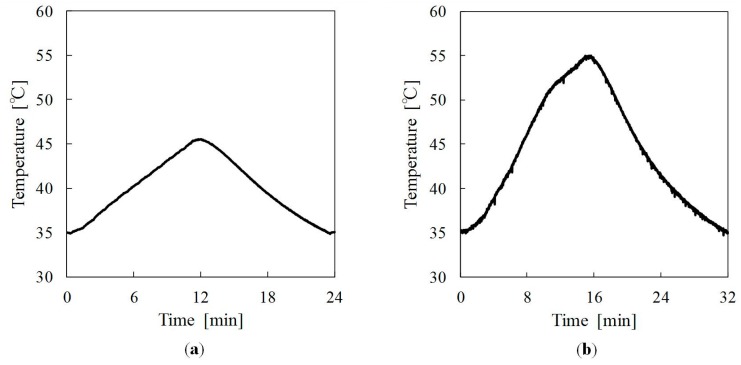
Surface temperature of green composite during one thermal cycling: (**a**) 35–45 °C; (**b**) 35–55 °C.

Thermal cycling tests of jute fiber, PLA, and green composite were conducted. Temperature ranges of thermal cycling tests were, respectively, 35–45 °C and 35–55 °C. As conditions of temperatures of 35–45 °C, the heating time was 12 min; the cooling time was 12 min. As conditions of temperatures of 35–55 °C, the heating time was 16 min; the cooling time was 16 min. The maximum number of cycles was 10^3^ cycles. Figure 2 shows surface temperatures of the green composite during one thermal cycling. Thermal cycling tests were conducted using a thermocouple and desk-top type high-temperature chamber (ST-120, Espec Co., Ltd., Kita-ku, Osaka, Japan).

### 3.2. Thermal Mechanical Anaysis

After thermal cycling tests, thermal mechanical analysis of PLA was conducted based on the Japanese Industrial Standard (JIS) K 7197 [34]. The PLA specimen was 12 mm long, 5 mm wide and 0.3 mm thick. Thermal mechanical analysis of jute fiber was also conducted after thermal cycling testing. Gauge lengths of jute fiber and PLA were 10 mm. The test load was 0.1 N. The temperature range was 30–160 °C. The heating rate was 5 °C/min. The numbers of specimens for jute fiber and PLA were, respectively, 10 and 5. Thermal mechanical analyses of jute fiber and PLA were conducted using a themomechanical analyzer (TMA-60, Shimazu Co., Ltd., Nakagyo-ku, Kyoto, Japan).

### 3.3. Quasi-Static Tensile Test after Thermal Cycling

After thermal cycling tests, quasi-static tensile tests of PLA and green composite were conducted respectively based on JIS K 7162 [35] and JIS K 7054 [36]. As a condition, the crosshead speed was 1 mm/min. Gauge lengths of PLA and green composite were 25 mm. The environmental temperature was RT. Numbers of specimens for green composite and PLA were, respectively, three. After quasi-static tensile tests, the fracture surface of the green composite was observed using scanning electron microscope (VE-7800, KEYENCE Co., Ltd., Higashiyodogawa-ku, Osaka, Japan).

Quasi-static tensile tests of jute fiber were conducted after thermal cycling tests of 30 specimens. The gauge length was 10 mm. The crosshead speed was 1 mm/min. The environmental temperature was RT. The cross sectional area at break point of jute fiber was estimated by elliptical approximation using a photograph of the jute fiber before quasi-static tensile testing. The tensile strength of jute fiber was calculated using a Weibull distribution. The Young’s modulus of jute fiber was calculated using the cross-sectional area at seven points. Quasi-static tensile tests of jute fiber, PLA, and green composite were conducted using a tensile testing machine (TENSILON RTG-1250A, A & D Co., Ltd., Toshima-ku, Tokyo, Japan).

## 4. Results and Discussion

### 4.1. Thermal Mechanical Analyses of Jute Fiber amd PLA after Thermal Cycling

**Figure 3:**
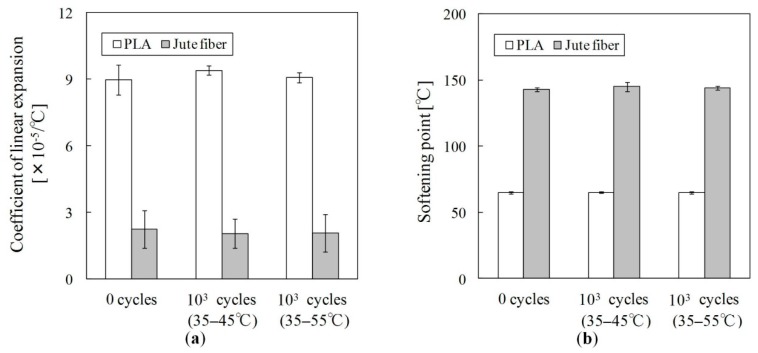
Thermal mechanical analyses of jute fiber and PLA after thermal cycling: (**a**) Coefficient of linear expansion; (**b**) Softening point.

Figure 3 shows effects of thermal cycling on the coefficient of linear expansion and softening points of jute fiber and PLA. In cases of temperatures of 35–45 °C and 35–55 °C, the coefficient of linear expansion of jute fiber and PLA at 10^3^ cycles remained almost unchanged in comparison with that of jute fiber and PLA at 0 cycles. Based on their results, coefficients of linear expansions of jute fiber and PLA were unaffected because thermal degradations of jute fiber and PLA did not occur during thermal cycling. The coefficient of linear expansion of jute fiber was 78% lower than that of PLA. Jute fiber consists mainly of cellulose. The coefficient of linear expansion of cellulose was 1.4 × 10^−5^ [1/°C] [37]. The coefficient of linear expansion of jute fiber was lower than that of PLA because of the cellulose in constituent materials.

In cases of temperatures of 35–45 °C and 35–55 °C, softening points of jute fiber and PLA did not change with an increase in the number of cycles. The softening point of jute fiber was higher than that of PLA. Softening points of lignin, hemicellulose, and cellulose in constituent materials of jute fiber are, respectively, 127–235 °C, 167–217 °C, and 231–253 °C [38]. The softening point of jute fiber was affected mainly by the softening point of lignin as its constituent material.

### 4.2. Tensile Properties of Jute Fiber, PLA, and Green Composite during Thermal Cycling

**Figure 4:**
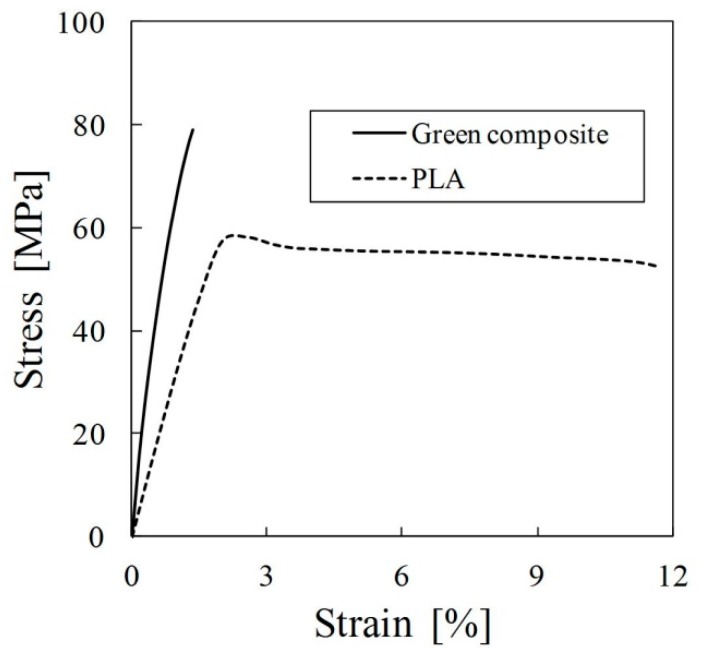
Stress-strain curves of green composite and PLA at 0 cycles.

Figure 4 shows stress-strain curves of green composite and PLA at 0 cycles. The stress-strain curve of the green composite was almost linear. The stress-strain curve of PLA was linear up to strain of 2%. Subsequently the stress-strain curve of PLA decreased slightly until strain 3%. The stress-strain curve of PLA remained almost unchanged at 4% or more. The average tensile strength of green composite and PLA at 0 cycles were, respectively, 79 MPa and 59 MPa. The average Young’s modulus of the green composite and PLA at 0 cycles were, respectively, 9 GPa and 3 GPa.

**Figure 5:**
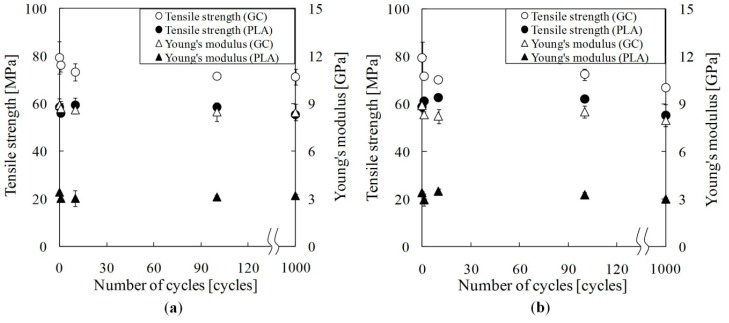
Tensile properties of green composite and PLA during thermal cycling: (**a**) 35–45 °C; (**b**) 35–55 °C.

Figure 5 shows tensile properties of green composite and PLA during thermal cycling. Furthermore, Table 1 shows tensile test results of green composite and PLA. For temperatures of 35–45 °C, the tensile strength of the green composite at one cycle decreased 4% in comparison with that of the green composite at 0 cycles. Subsequently, the tensile strength of green composite decreased gradually with an increased number of cycles. The tensile strength of the green composite at 10^3^ cycles decreased by 10% in comparison with that of the green composite at 0 cycles. Young’s modulus of green composite decreased slightly with an increased number of cycles.

For temperatures of 35–55 °C, the tensile strength and Young’s modulus of the green composite at one cycle decreased 9% and 7%, respectively, in comparison with that of green composite at 0 cycles. Subsequently, the tensile strength and Young’s modulus of green composite remained almost unchanged until 10^2^ cycles. Tensile strength and Young’s modulus of green composite at 10^3^ cycles decreased respectively 7% and 6% in comparison with those of green composite at 10^2^ cycles. The tensile strength and Young’ modulus of the green composite at temperatures of 35–55 °C was lower than those of green composite at temperatures of 35–45 °C when thermal cycling tests were conducted until 10 cycles. In the case of 10^3^ cycles, tensile strength and Young’ modulus of green composite at temperatures of 35–55 °C was lower than those of the green composite at temperatures of 35–45 °C.

When thermal cycling tests of PLA were conducted at temperatures of 35–45 °C and 35–55 °C, the tensile strength and Young’s modulus of PLA remained almost unchanged except for tensile strength at temperatures of 35–55 °C.

**Figure 6:**
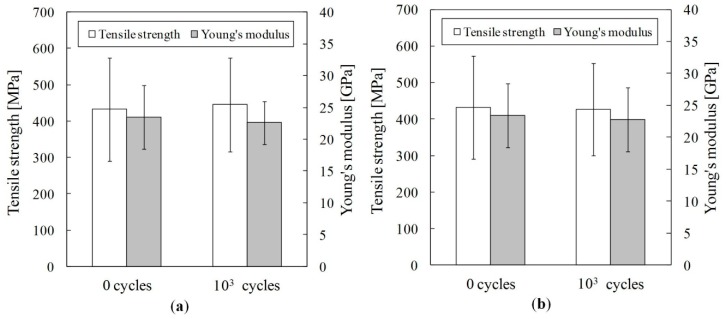
Tensile property of jute fiber after thermal cycling: (**a**) 35–45 °C; (**b**) 35–55 °C.

Figure 6 presents tensile properties of jute fiber after thermal cycling. For temperatures of 35–45 °C and 35–55 °C, the tensile strength and Young’ modulus of jute fiber remained almost unchanged after thermal cycling. Their results implied that the tensile property of jute fiber during thermal cycling was only slightly affected because of the heat resistance of the jute fiber constituent materials.

As presented in the results above, the tensile properties, coefficient of linear expansion and softening point of jute fiber and PLA remained almost unchanged during thermal cycling tests of jute fiber and PLA. However, tensile properties of the green composite at 10^3^ cycles were lower than those of green composite at 0 cycles when thermal cycling tests were conducted at temperatures of 35–45 °C. In addition, expansion and contraction of the jute fiber and PLA in the green composite are related to the temperature range and the coefficient of linear expansion of jute fiber and PLA. Tensile properties of the green composite at an early stage were decreased because of the expansion and contraction of jute fiber and PLA by the increase of the temperature range. Therefore, tensile properties of the green composite during thermal cycling were affected by a difference of coefficient of linear expansion of jute fiber and PLA.

### 4.3. Fracture Surface of Green Comsposite after Thermal Cycling

**Figure 7:**
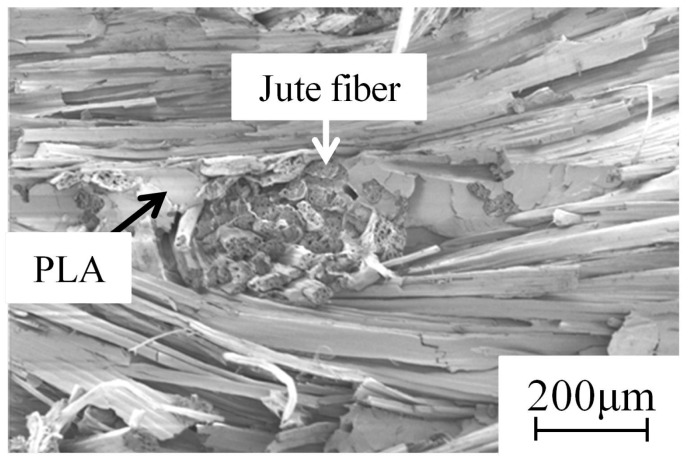
Fiber pull out in fracture surface of green composite at 0 cycles.

**Figure 8:**
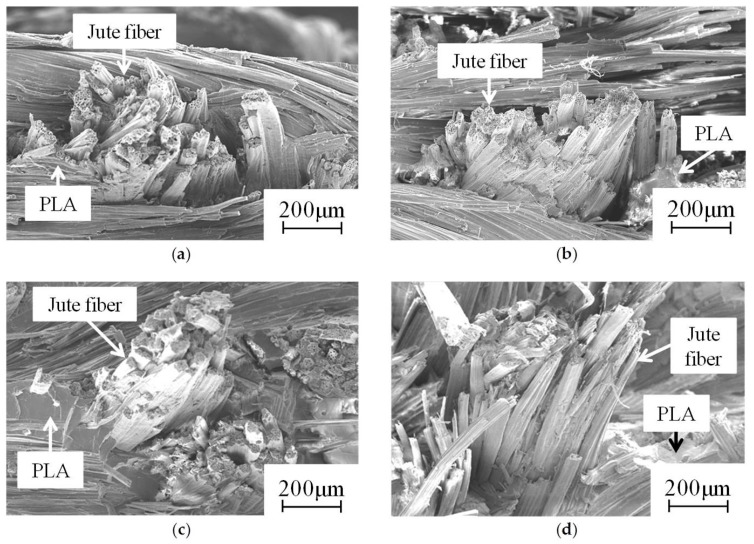
Fiber pull out in fracture surface of green composite after thermal cycling (35–45 °C): (**a**) 1 cycle; (**b**) 10 cycles; (**c**) 10^2^ cycles; (**d**) 10^3^ cycles.

**Figure 9:**
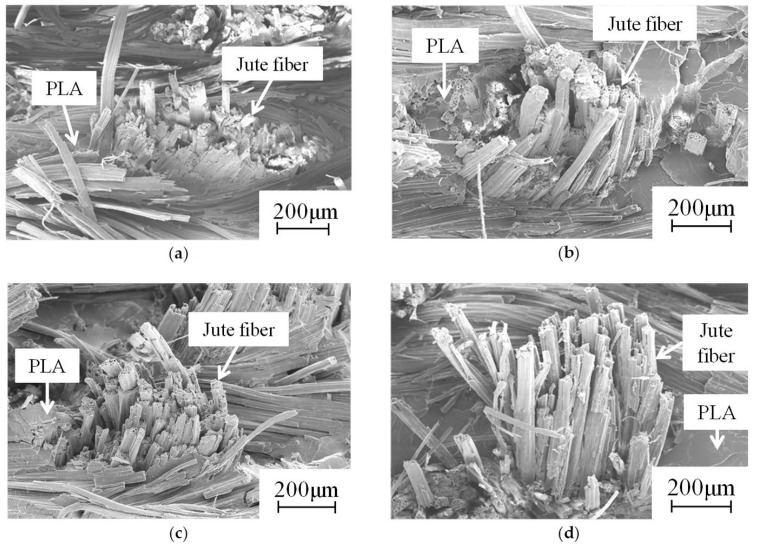
Fiber pull out in fracture surface of green composite after thermal cycling (35–55 °C): (**a**) 1 cycle; (**b**) 10 cycles; (**c**) 10^2^ cycles; (**d**) 10^3^ cycles.

Figure 7 presents the fracture surface of the green composite at 0 cycles. Figure 8 and Figure 9 show fracture surfaces of green composites after thermal cycling. Fiber pull out in fracture surfaces of green composites were found before and after thermal cycling. For temperatures of 35–45 °C, the lengths of fiber pull out in fracture surfaces of green composites at one cycle and 10 cycles were longer than that of green composite at 0 cycles. After 10 cycles, the length of fiber pull out in the fracture surface of the green composite at 10^2^ cycles increased slightly. In addition, the length of fiber pull out in the fracture surface of the green composite at 10^3^ cycles became great.

For temperatures of 35–55 °C, the length of fiber pull out in the fracture surface of the green composite at one cycle was longer than that of the green composite at 0 cycles. Subsequently, the length of the fiber pull out in the fracture surface of the green composite remained almost unchanged until 10^2^ cycles. The length of fiber pull out of fracture surface of green composite at 10^3^ cycles was longer than that of the green composite at 10^2^ cycles.

The coefficient of linear expansion of jute fiber was 78% lower than that of PLA. Interfacial adhesion between the fiber and resin during thermal cycling was decreased, probably because of expansion and contraction by the difference of the coefficient of linear expansions of jute fiber and PLA. Interfacial adhesion between the fiber and resin during thermal cycling was greatly decreased by an increase of the temperature range. In addition, debonding at the interface between the fiber and resin during thermal cycling might occur because of the decrease of interfacial adhesion between fiber and resin for temperatures of 35–55 °C. Therefore, the tensile property of green composites was affected because of the decreased interfacial adhesion between the fiber and resin under thermal cycling.

## 5. Conclusions

In this study, residual tensile properties of plain woven jute fiber reinforced PLA were investigated during thermal cycling. Results suggest the following conclusions.

For temperatures of 35–45 °C, the tensile strength of the green composite decreased gradually with an increased number of cycles. Young’s modulus of the green composite decreased slightly with an increased number of cycles. For temperatures of 35–55 °C, the tensile strength and Young’s modulus of the green composite at one cycle was lower than that of green composite at 0 cycles. Subsequently tensile strength and Young’s modulus of green composite remained almost unchanged until 10^2^ cycles. The tensile strength and Young’s modulus of green composite at 10^3^ cycles were lower than those of the green composite at 10^2^ cycles. For temperatures of 35–45 °C and 35–55 °C, tensile properties, coefficients of linear expansion and softening points of jute fiber and PLA did not change with an increased number of cycles. However, the coefficient of linear expansion of jute fiber was lower than that of PLA. Interfacial adhesion between the fiber and resin during thermal cycling was decreased, probably because of expansion and contraction by the different coefficients of linear expansion of jute fiber and PLA. In addition, debonding at the interface between the fiber and resin might have occurred because of the increase of the temperature range. Therefore, tensile properties of green composites were affected because of the decrease of interfacial adhesion between the fiber and resin during thermal cycling.

## Figures and Tables

**Table 1:** Tensile test results of green composite and PLA (AVG: average value, SD: standard division).

Material	Temperature Range	Cycle	Young’s Modulus (GPa)		Tensile Strength (MPa)		Failure Strain (%)	
			AVG	SD	AVG	SD	AVG	SD
Green composite	–	0	8.9	0.4	79	6.8	1.5	0.1
	35–45 °C	1	8.7	0.4	76	2.7	1.3	0.1
		10	8.6	0.2	73	3.6	1.3	0.1
		100	8.5	0.6	72	0.5	1.2	0.1
		1000	8.5	0.5	71	3.4	1.3	0.1
	35–55 °C	1	8.3	0.2	72	1.3	1.4	0.1
		10	8.2	0.4	70	1.3	1.4	0.1
		100	8.5	0.4	72	2.5	1.4	0.1
		1000	8.0	0.4	67	1.3	1.3	0.2
PLA	–	0	3.4	0.1	59	0.3	12	3.5
	35–45 °C	1	3.0	0.2	56	0.7	9.0	1.2
		10	3.0	0.5	60	2.9	12	4.2
		100	3.1	0.1	58	1.7	9.0	3.0
		1000	3.2	0.1	55	1.3	8.7	2.3
	35–55 °C	1	3.0	0.4	61	0.1	16	1.4
		10	3.5	0.1	63	0.6	12	3.2
		100	3.3	0.2	62	1.0	13	2.1
		1000	3.0	0.1	55	4.7	8.4	2.0
